# Plastic response to early shade avoidance cues has season‐long effect on *Beta vulgaris* growth and development

**DOI:** 10.1111/pce.14171

**Published:** 2021-08-29

**Authors:** Albert T. Adjesiwor, Joseph G. Ballenger, Cynthia Weinig, Brent E. Ewers, Andrew R. Kniss

**Affiliations:** ^1^ Department of Plant Sciences University of Wyoming Laramie Wyoming USA; ^2^ Department of Botany University of Wyoming Laramie Wyoming USA; ^3^ Department of Molecular Biology University of Wyoming Laramie Wyoming USA; ^4^ Program in Ecology University of Wyoming Laramie Wyoming USA; ^5^ Present address: Kimberly Research and Extension Center University of Idaho Kimberly 83341 ID USA

**Keywords:** environmental plasticity, light quality, weed competition, weed removal timing, yield potential

## Abstract

Early‐emerging weeds are known to negatively affect crop growth but the mechanisms by which weeds reduce crop yield are not fully understood. In a 4‐year study, we evaluated the effect of duration of weed‐reflected light on sugar beet (*Beta vulgaris* L.) growth and development. The study included an early‐season weed removal series and a late‐season weed addition series of treatments arranged in a randomized complete block, and the study design minimized direct resource competition. If weeds were present from emergence until the two true‐leaf sugar beet stage, sugar beet leaf area was reduced 22%, leaf biomass reduced 25%, and root biomass reduced 32% compared to sugar beet grown season‐long without surrounding weeds. Leaf area, leaf biomass, and root biomass was similar whether weeds were removed at the two true‐leaf stage (approximately 330 GDD after planting) or allowed to remain until sugar beet harvest (approximately 1,240 GDD after planting). Adding weeds at the two true‐leaf stage and leaving them until harvest (~1,240 GDD) reduced sugar beet leaf and root biomass by 18% and 23%, respectively. This work suggests sugar beet responds early and near‐irreversibly to weed presence and has implications for crop management genetic improvement.

## Abstract 2

The presence of weed‐reflected light during sugar beet emergence through the two true‐leaf stage (~16 d after emergence) had a more detrimental impact on crop growth than the same environmental cue beginning at the two true‐leaf stage until harvest 50 d later. Sugar beet responded irreversibly to the presence of early‐season weed presence, and therefore shade avoidance may be a major contributor to sugar beet yield loss due to weeds.

## 1 INTRODUCTION

Plants are exposed to heterogeneous environments comprising different levels of stress and competition for resources. As sessile organisms, plants often exhibit morphological plasticity in response to adverse environmental conditions. Many of these responses involve developmental trade‐offs that affect plant growth and development and their ability to respond to future environmental cues (Carriedo, Maloof, & Brady, 2016; Huber, Nieuwendijk, Pantazopoulou, & Pierik, 2021; Wille, Pipper, Rosenqvist, Andersen, & Weiner, 2017).

Mechanisms described as optimality, balanced growth or functional equilibrium suggest that plants allocate more resources to the organ(s) which is(are) acquiring the resource that is currently the most limiting (Gedroc, McConnaughay, & Coleman, 1996; Poorter et al., 2012; Shipley & Meziane, 2002). Plants will allocate more resources to root growth if the limiting factor is below‐ground (e.g., nutrients, water) and more resources to shoot growth if the limiting factor is light (Fazlioglu, Al‐Namazi, & Bonser, 2016; Freschet, Violle, Bourget, Scherer‐Lorenzen, & Fort, 2018). The ecological advantage of this functional response is clear; by allocating more resources to the plant organ acquiring the most limiting resource, plants acquire the needed resources for growth and reproduction. However, the timing of these responses is critical. For example, plants subjected to moderate levels of moisture stress show little to no changes in size and root mass. However, once plants are subjected to severe moisture stress biomass is reduced by >50%, and relative allocation to roots increases strongly at the expense of shoot growth (Padilla, Miranda, Jorquera, & Pugnaire, 2009). Plants might not respond very strongly to relatively short periods of moisture stress because of the unpredictability of precipitation. Increasing allocation to roots too quickly might result in suboptimal growth after the restoration of the water supply (Poorter et al., 2012). This is also true for other environmental responses, such as stem elongation responses to light cues of neighbour proximity (Bongers et al., 2019; de Wit et al., 2018; Huber et al., 2021).

One particularly important environmental cue with economic consequences, that is, early allocation versus later growth trade‐offs, is shade. In dense plant canopies such as weedy crop fields, the plant environment is enriched with far‐red (FR) light due to reflected FR by green vegetation. For example, the ratio of reflected red (R) to FR was 0.06 when *B. vulgaris* was surrounded by common lambsquarters (*Chenopodium album* L.) compared to 0.7 when surrounded by bare soil (Adjesiwor & Kniss, 2020). With the aid of a family of photoreceptors, particularly cryptochromes and phytochromes, plants can sense and respond to changes in R:FR and blue‐violet light in their surroundings (Casal, 2013). Cryptochromes are blue and ultra‐violet light receptors involved in the response to blue to green light ratios (cryptochrome 1) or photoperiod (cryptochrome 2). Phytochromes exist as either R absorbing phytochrome (Pr) or FR light absorbing phytochrome (Pfr). When the stable form of the phytochrome (Pr) absorbs R light (660 nm peak absorbing wavelength), it converts to Pfr. The conformation change is reversible: under FR light (wavelengths neighbouring 730 nm), the Pfr form of the phytochrome converts back to the Pr form Hopkins (Hopkins & Hüner, 2008).

Our work presented here focussed solely on phytochrome‐mediated neighbour detection and subsequent plant allocation response. In the context of phytochrome‐mediated neighbour detection, reduced R:FR is sensed by phytochrome B, which then binds to phytochrome interacting factors (PIFs) that are then ubiquitinated and degraded by the COP‐1 complex (Leivar & Quail, 2011; Pham, Kathare, & Huq, 2018). This results in large‐scale transcriptional regulation, which ultimately results in a series of developmental changes that serve to circumvent being shaded by other plants. These changes are collectively referred to as shade avoidance syndrome ((Ballaré & Pierik, 2017; Pantazopoulou, Bongers, & Pierik, 2021; Roig‐Villanova & Martínez‐García, 2016).

The developmental changes set off by shade avoidance response (SAR) include differential allocation to roots, seeds, and leaves that are important in crop production (Page, Tollenaar, Lee, Lukens, & Swanton, 2010; Schambow, Adjesiwor, Lorent, & Kniss, 2019). When these effects are combined, crops exhibiting the shade avoidance syndrome in response to weeds constitute a potentially significant source of yield loss in agriculture (Huber et al., 2021; Page et al., 2012; Wille et al., 2017). This has been demonstrated in seed crops like maize (*Zea mays* L.) and soybean (*Glycine max* (L.) Merr.) (Green‐Tracewicz, Page, & Swanton, 2011, 2012; Liu, Mahoney, Sikkema, & Swanton, 2009). However, *B. vulgaris* is a unique plant in that it is a biennial usually cultivated as an annual for its sucrose‐rich enlarged roots. Thus, SAR in *B. vulgaris* could be substantially different than seed crops like maize and soybean. We have previously shown that leaf number, leaf area, and biomass is reduced by season‐long shade avoidance cues in three different sub‐species of *B. vulgaris* (Schambow et al., 2019). In the current study, we experimentally manipulated the duration of the shade avoidance cue to assess the mechanism by which *B. vulgaris* growth is reduced. Specifically, we wanted to test whether shade avoidance cues during the early and late stages of *B. vulgaris* growth have the same effects on morphology and growth. It has long been known that early‐emerging weeds (weeds that emerge with the crop) have more detrimental impact on crop yield than late‐emerging weeds (Dew, 1972; O'Donovan, Remy, O'Sullivan, Dew, & Sharma, 1985; Swanton et al., 1999). In maize, 5% of potential yield is lost within 183 growing degree days (corresponding to 3–5 leaf stage) after planting due to weeds (Page et al., 2012).

It is logical to ascribe this phenomenon to the reduced crop growth from early‐season resource depletion from weed competition. However, root interaction and growth resource (e.g., water, nutrients and light) competition is minimal early in the growing season. Thus, growth reduction from early‐season weed presence could be due to other competitive effects of weeds rather than resource depletion. Rajcan, Chandler, and Swanton (2004) found that maize seedlings detected changes in light quality caused by the presence of grass (which was used to simulate low‐growing weeds) and responded by adjusting carbon allocation and leaf orientation to optimize the light interception. Studies have shown that stem and petiole extension and hyponasty are among the most common SARs (Cerrudo et al., 2017; Franklin & Whitelam, 2005; Yang & Li, 2017). We hypothesized that: (a) early‐season reflected light from neighbouring weeds would induce hyponasty and increase allocation to petiole extension growth, thereby reducing root biomass of sugar beet; and (b) the timing and duration of the shade avoidance cues would influence the severity of the observed SAR. To test this hypothesis, we used a study design that prevents any direct resource competition, making it possible to assess the sole effect of reflected light on sugar beet growth. The understanding of shade avoidance responses in sugar beet and the role shade avoidance cues plays in yield loss during weed competition could provide novel insights into the critical period for weed control in this and other crops.

## 2 MATERIALS AND METHODS

### 2.1 Experimental site and design

Field and greenhouse studies were conducted from 2015 to 2019 at the University of Wyoming Laramie Research and Extension Center (LREC), Laramie, WY. The study comprised 12 weed removal or addition timing (growing degree days [GDD]) treatments in 2015 and 2016 and nine treatments in 2018 and 2019 (Table 1). The study was also conducted in 2017, but severe weather events and an irrigation system failure led to uneven emergence and heavy losses of experimental units, so the 2017 data were excluded from the analysis.

**TABLE 1:** Treatment descriptions showing the start and end of weed treatments in growing degree days (GDD)

Timing	Start of weed treatment				End of weed treatment			
	2015	2016	2018	2019	2015	2016	2018	2019
Early series^a^	0	0	0	0	330	297	365	352
	0	0	0	0	398	384	565	552
	0	0	0	0	462	451	765	752
	0	0	0^b^	0^b^	532	511	1,240^b^	1,223^b^
	0	0	‐	‐	582	581	‐	‐
	0	0	‐	‐	631	648	‐	‐
	0^a^	0^a^	‐	‐	1,181^b^	1,185^b^	‐	‐
Late series^c^	330	297	365	352	1,181	1,185	1,240	1,223
	398	384	465	452	1,181	1,185	1,240	1,223
	462	451	565	552	1,181	1,185	1,240	1,223
	631	648	765	752	1,181	1,185	1,240	1,223
	1,181^d^	1,185^d^	1,240^d^	1,223^d^	1,181^d^	1,185^d^	1,240^d^	1,223^d^

^a^ Early series treatments denote weed presence at sugar beet emergence.

^b^ Late series treatments weed addition after sugar beet emergence.

^c^ Weed presence from emergence to harvest (season‐long weed presence).

^d^ Season‐long weed‐free treatment.

**FIGURE 1:**
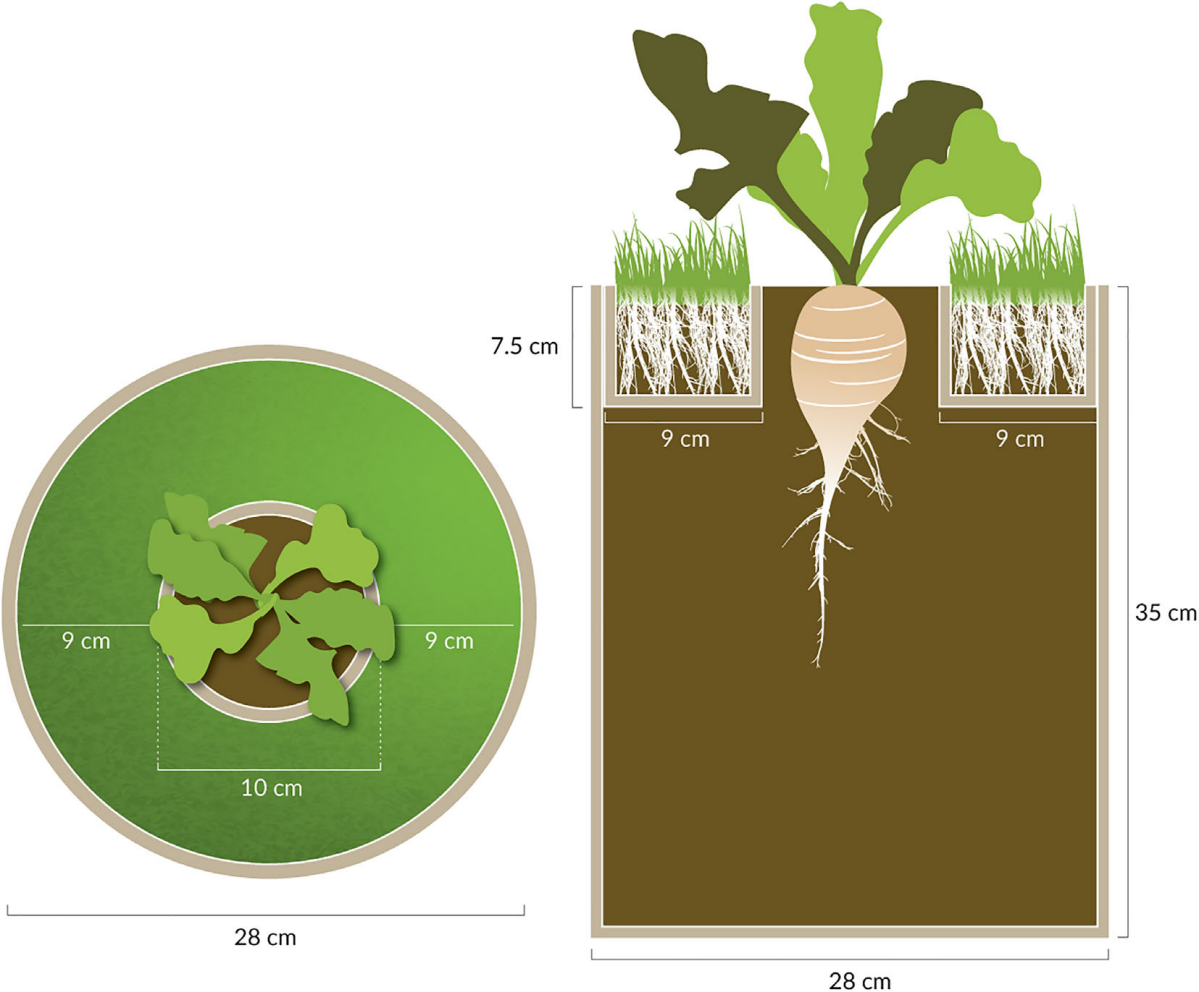
Illustration of the grass treatment used in the field experiment from the top‐view (left) and a cross‐section (right), modelled after (Green‐Tracewicz et al., 2011) as modified by (Schambow et al., 2019). Sugar beet was planted into the centre ring, and was allowed to grow using the full depth (35 cm) of the 21‐L pail. Grass roots were constrained to the top 7.5 cm and outer 9 cm of the pail, and were isolated from the sugar beet roots using plastic. Grass was clipped as needed to minimize any direct shading of the sugar beet plant in the centre. For the soil treatment, the design was the same, except no grass was planted into the potting media in the outer ring. Drawing by Jessica Perry

The large‐pail design used by Green‐Tracewicz et al. (2011) and modified by Schambow et al. (2019) was used in these studies (Figure 1). This approach prevents any direct resource competition, making it possible to assess the sole effect of reflected light on sugar beet growth. Briefly, 19 L black plastic pails were filled with a potting mix (Berger, BM Custom Blend, Saint‐Modeste, QC Canada) leaving about 7.5 cm head‐space. A cardboard tube (10 cm × 122 cm Staples Kraft Heavy‐Duty Mailing Tube: Staples Inc., Framingham, MA) of height 7.5 cm and 10 cm diameter was taped (Black Gorilla Tape: Gorilla Glue Co., Cincinnati, Ohio, USA) to a 2‐ml white plastic bag and laid on top of the potting mix such that the cardboard tube was in the middle of the pail and flush with the rim of the pail. The plastic prevented roots of the weed from interacting with sugar beet roots (Figure 1). Potting mix was then added onto the plastic and into the cardboard tube.

Three sugar beet seeds (cultivar ‘BTS60RR27’ in 2015 and 2016 and ‘RR014GEM50’ in 2018 and 2019) were planted per pail in the centre of the cardboard tube on 3 July 2015; 31 May 2016; 1 June 2018; and 3 June 2019. Cultivars were chosen solely based on their commercial availability – these cultivars were widely grown in the region. Genetic background between cultivars was unlikely to have a substantial impact on our results; we have previously documented similar season‐long shade avoidance responses in three separate *B. vulgaris* sub‐species of sugar beet, table beet and Swiss chard (Schambow et al., 2019). Emerged seedlings were thinned to one seedling per pail immediately after emergence. Sod of Kentucky bluegrass (*Poa pratensis* L.) was used to simulate low‐growing weeds and planted around the cardboard tube in the weedy treatments (Figure 1).

The treatments were grouped into early‐season removal and late‐season addition treatment series (Table 1). The early‐season removal series included treatments with weeds present at sugar beet emergence and then removed later in the growing season. Standard weed management practices in sugarbeet include weed removal (usually with a herbicide) at the early two‐true leaf stage of development, and our earliest removal timing in this study coincides with that practice. The late‐season addition series included treatments in which weeds were added at various times during the season and allowed to remain until sugar beet harvest. Treatments were arranged in a randomized complete block with 15 blocks in 2015 and 2016 and 27 blocks in 2018 and 2019.

**FIGURE 2:**
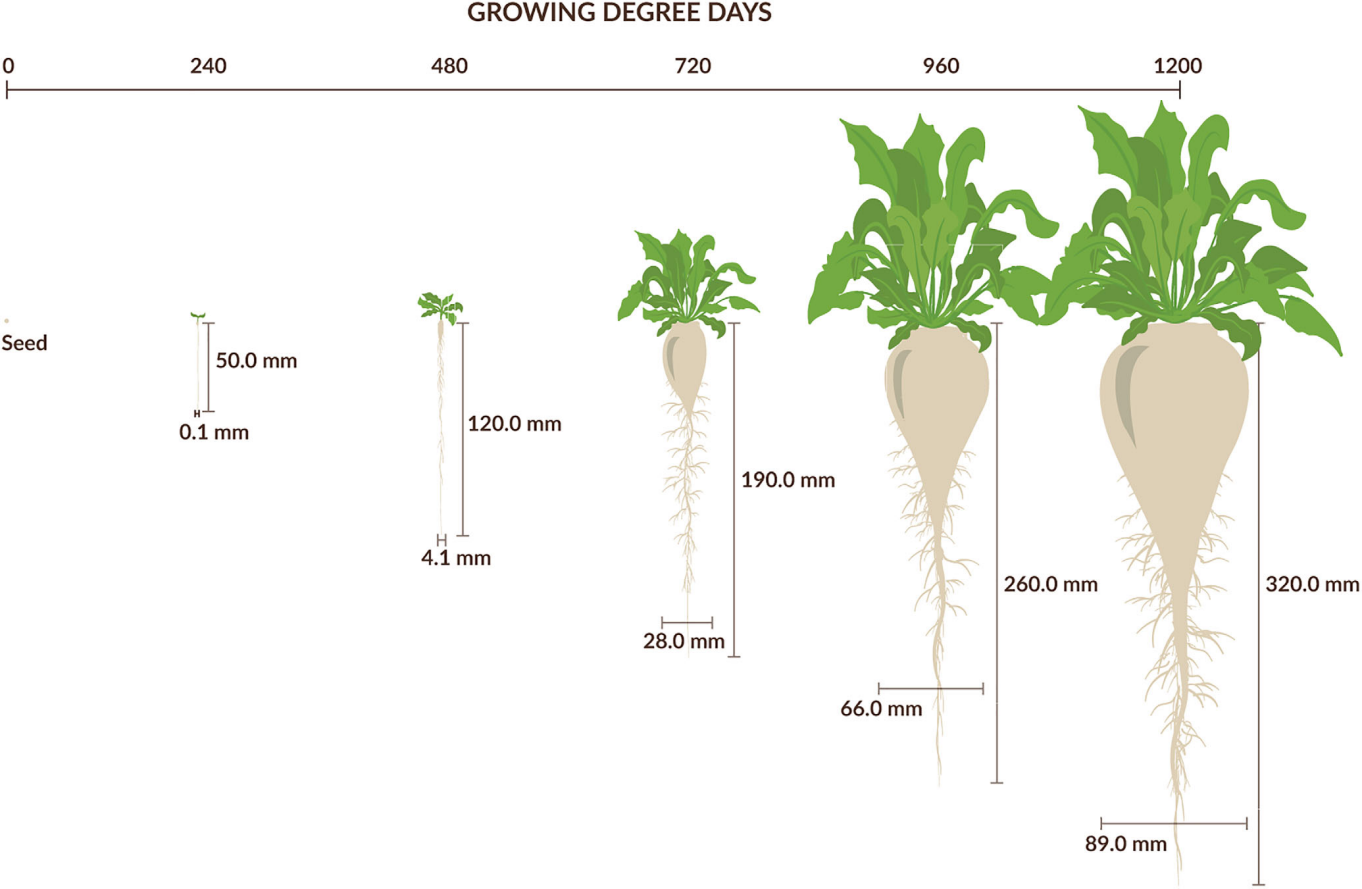
Typical sugar beet growth progression during the growing season. Growing degree days (GDD) calculated using the parameters described in Equation (1). The 240, 480, 720, 920 and 1,200 GDD correspond to approximately 15, 30, 45 and 75 days after planting sugar beet. Drawing by Jessica Perry [Colour figure can be viewed at wileyonlinelibrary.com]

Growing degree days (GDD) were estimated from daily minimum and maximum temperature [Equation (1)], where *T*_max_ is the daily maximum temperature (°C), *T*_min_ is the daily minimum temperature (°C) and *T*_base_ is the base temperature of 1.1°C (Holen & Dexter, 1997; NDAWN Center, 2020). The basic concept of GDD is that crop development will only occur when the temperature exceeds a certain minimum threshold (1.1°C for sugar beet), and increases linearly with temperature above the base until a maximum is reached (McMaster & Wilhelm, 1997). The base temperature is determined experimentally and varies for each organism. Thus, GDD is a more reliable predictor of crop development than calendar days. The first weed removal (early‐season series) and first weed addition (late‐season series) timings corresponded to the sugar beets reaching the two true‐leaf stage, which occurred between 297 and 365 GDD after planting, depending on the year (Figure 2). Final harvest occurred between 1,181 and 1,240 GDD after sugar beet planting.

(1) $$\mathrm{GDD}=\left(\frac{T_{\max}+T_{\min}}{2}\right)-T_{\text{base}}.$$

Sugar beet was drip irrigated daily until maturity to ensure no yield limitation due to moisture stress. The model weed was also drip‐irrigated separately to ensure green growth and optimal reflection of FR light. Sugar beet was fertilized with 85 g pail^−1^ of 14:14:14:5.5% (N:P:K:S) polymer‐coated fertilizer (Florikote™ NPK, Florikan E.S.A.‐LLC, Sarasota, FL) at planting to ensure slow and continuous release of nutrients throughout the growing season. The model weed was clipped regularly to prevent direct competition for sunlight.

Using the same experimental system, a greenhouse study was conducted from 11 Nov 2018 to 5 Feb 2019. Day and night temperatures were maintained at 22 and 20°C, respectively. No supplemental light was used, and plants were overhead irrigated twice a day throughout the experiments. The greenhouse study did not include the addition or removal treatments and comprised only two treatments: sugar beet surrounded by grass or no grass (weed‐free), to enable detailed leaf measurements under controlled conditions.

### 2.2 Data collection

Number of leaves on each plant were counted weekly after seedling emergence. Leaf angle (from the soil horizon) of the oldest, healthiest leaf was measured using a protractor between 648 and 760 GDD (41 and 49 days after planting), depending on the year. The angle of the oldest leaves was measured because it was not possible to measure the angle of younger leaves without breaking the older leaves. In addition, the angle of the younger leaves depends on the angle or position of the older leaf. Thus, the angle of the healthy older leaf provided a good estimate of the plant leaf angle. Sugar beet plants from the field study were harvested on 16 September 2015; 10 August 2016; 14 August 2018; and 19 August 2019. At harvest, leaves were separated from the roots, and roots were washed to remove potting media. Leaves were counted, and total leaf area per plant was measured by feeding leaves through an LI‐3100C (LI‐COR Inc., Lincoln, Nebraska, USA), respectively. Leaves were then dried at 60°C for 48 hr and weighed to obtain shoot biomass per plant. Roots were sliced (to speed drying) and dried for 72 hr at 60°C then weighed to obtain root biomass per plant.

In the greenhouse study, plants were harvested on 5 Feb 2019, and the leaves were separated from the crown according to the order of appearance or developmental stage (i.e., according to phyllotaxy). The oldest leaf pair were assigned position numbers one and two, the second oldest leaf pair assigned position numbers three and four, and so forth. Thus, a lower leaf position corresponds to an older leaf and higher leaf position corresponds to a younger leaf at the time of harvest. Length of each leaf and petiole was measured using a ruler, and leaf width (at the widest part of the lamina) and leaf area were measured using a leaf area meter (LI‐3100C, LI‐COR Inc., Lincoln, Nebraska, USA).

### 2.3 Data analyses

For each response variable, a linear mixed‐effects model and nonlinear mixed‐effects model (4‐parameter log‐logistic function) was compared, and the optimal model was selected based on Akaike's Information Criterion (AIC). In all cases (except for leaf angle), the linear model provided a similar (AIC values within 10) or better (linear model AIC < nonlinear model AIC) fit to the data than the nonlinear model.

The nonlinear model used to describe leaf angle was of the form

$$f\left(x\right)=c+\frac{d-c}{1+e^{b\left(\log\left(x\right)-\log\left(e\right)\right)}},$$

where *x* is the duration of weedy (early‐season removal series) or weed‐free (late‐season addition series) GDD after planting; *f*(*x*) is the leaf angle as a function of GDD after planting; *d* and *c* are upper and lower limits, respectively, indicating the estimated leaf angle at extreme values of *x*; *b* is the slope at the inflection point of the curve; and *e* is the number of GDD at which the inflection point occurs. The medrc package (ver. 1.1–0, Gerhard & Ritz, 2016) in the R statistical language was used to fit nonlinear models, with random components included for the *c*, *d* and *e* parameters.

A linear mixed‐effects ANOVA was performed in R statistical language (ver. 4.0.4) using the lmer function of the lme4 package (ver. 1.1–23) and convenience functions from the lmerTest package (ver. 3.1–2) (Bates, Maechler, Bolker, & Walker, 2015; Kuznetsova, Brockhoff, & Christensen, 2017; R Core Team, 2020). Weed removal timing (early‐season series) or addition timing (late‐season series) were considered fixed effects, and year was considered a random effect. A weed‐free indicator variable (*wf*) was included in the linear model, given a value of 1 for the weed‐free treatment or a value of 0 if the treatment contained weeds at any point during the season; each fixed‐effect (linear effect of addition or removal, and the weed‐free indicator) was dropped from the model and the full and reduced models were compared with an F‐test. If removal of a fixed‐effect resulted in a significant reduction in model fit compared to the full model (*p* < .1), then that effect was retained in the final model. In this way, if there was a significant effect of the *wf* indicator variable, but no significant effect of the removal or addition timing, then the effect was assumed to be attributable to only the presence of weeds during early sugar beet development, and not to the duration of weed presence beyond that period. Conversely, if there was an effect of addition or removal timing, but no effect of the *wf* indicator variable, then the response was attributed to the linear duration of weed presence. If both effects were retained, then it suggests a discontinuous effect, where duration of weed presence influenced the response, but a greater (or lesser) response was attributable to the early development stage.

Two‐sample t‐tests were used to compare the season‐long weed‐free treatment to season‐long weedy treatments in the greenhouse study for final number of leaves, number of senesced leaves at harvest and plant biomass. A non‐parametric local regression (loess) was fit to the petiole proportion of leaf length as a function of leaf age for the greenhouse study. The 95% confidence intervals at each leaf pair were used as a conservative estimate of statistical difference between the season‐long weed‐free versus season‐long weedy treatments (Austin & Hux, 2002).

## 3 RESULTS

### 3.1 Shade avoidance cues at sugar beet emergence and the duration of shade avoidance cues added later affected sugar beet growth and development

When the shade avoidance cue was present at sugar beet emergence (early‐season removal series), the effect of the weed‐free indicator variable (*p* = .065) and the linear effect of weed duration (*p* < .001) impacted sugar beet leaf number at harvest (Table 2). However, reductions in sugar beet leaf area, leaf biomass and root biomass were attributable to the *wf* indicator variable, indicating presence of weeds through the two true‐leaf stage caused most of the observed response (*p* < .001). Once adjusted for the weed‐free versus weedy treatment comparison, there was no effect of weedy duration in the early‐season removal series (*p* > .42), suggesting the duration of weed presence beyond the two true‐leaf stage had minimal effect. In contrast, when the shade avoidance cue was added after the sugar beet two true‐leaf stage, duration of weed presence had a significant effect on sugar beet growth reduction.

**TABLE 2:** Model selection *p*‐values for sugar beet leaf and root measurements from the field study

Response variable	Treatment series	*p*‐value	
		Weed‐free versus weedy (*wf* indicator variable)	Weedy duration
Leaf number	Early‐season removal	**.065**	**<.001**
	Late‐season addition	.280	**<.001**
Leaf area	Early‐season removal	**<.001**	.704
	Late‐season addition	.975	**<.001**
Leaf biomass	Early‐season removal	**<.001**	.832
	Late‐season addition	.641	**<.001**
Root biomass	Early‐season removal	**<.001**	.420
	Late‐season addition	.748	**<.001**

*Note*: Null hypothesis was that removal of the fixed effect term (weedy vs. non, duration) does not reduce model fit; significant *p*‐values suggest the term should remain in the model. *p*‐values associated with final selected model terms are printed in bold.

### 3.2 Shade avoidance cues shortly after emergence irreversibly affect leaf development

**FIGURE 3:**
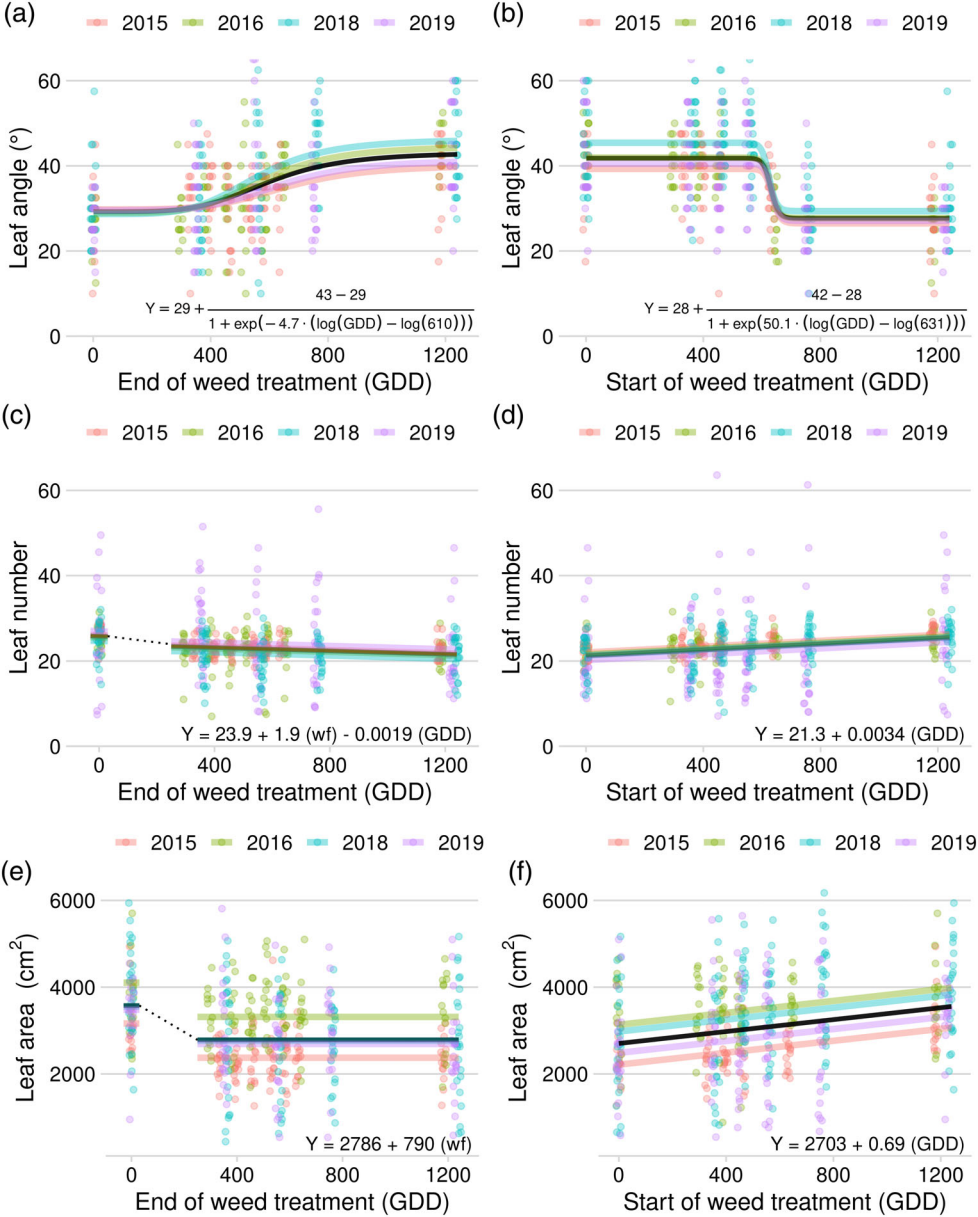
Relationship between duration of weed presence and leaf angle approximately 45 days after planting (a and b), number of sugar beet leaves at harvest (c and d), and leaf area at harvest (e and f) in a field study conducted from 2015 to 2019, Laramie, WY. Panels on the left (a, c and e) included treatments in which weeds were present at sugar beet emergence until removed later in the growing season (removal series). The dotted line in panel A indicates a break in the linear trend in the absence of the weed‐free treatment. Panels on the right (b, d and f) include treatments in which weeds were added at various times during the season and allowed to remain until harvest (addition series). Regression equation in each panel is for the fixed‐effects mean (black lines); ‘*wf*’ is an indicator variable for the weed‐free treatment (1 if weed‐free season‐long, 0 if weeds were present at any point during the season) [Colour figure can be viewed at wileyonlinelibrary.com]

**FIGURE 4:**
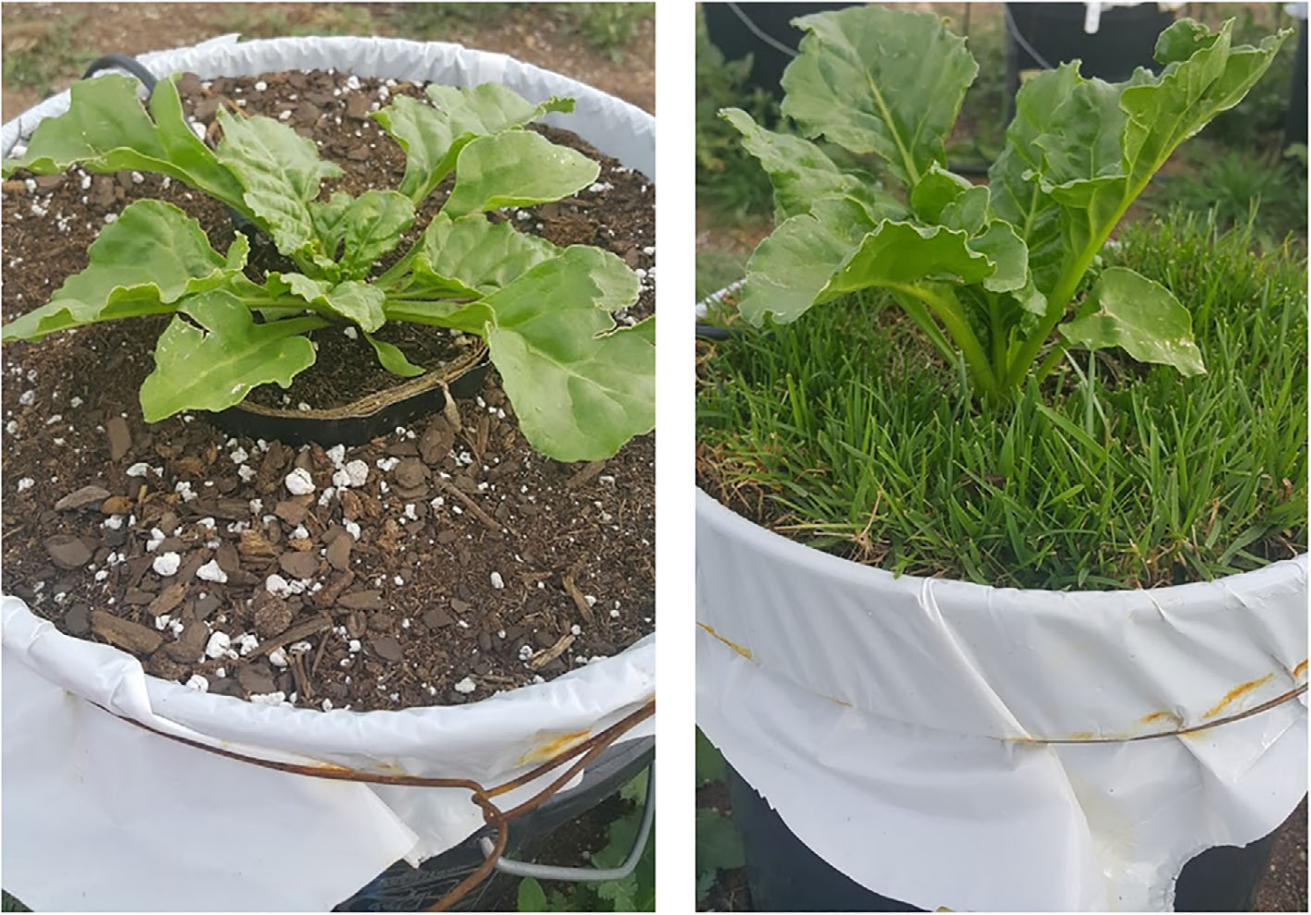
Sugar beet shoot growth in the weed‐free (left) and weedy (right) at 41 days (648 growing degree days) in the field experiment in 2016, Laramie WY. Diameter of the top of the bucket is 28 cm; diameter of the inner tube opening is 10 cm [Colour figure can be viewed at wileyonlinelibrary.com]

Sugar beet leaf angle was affected by the duration of weed presence (Figure 3a,b), although the relationship between duration of weed presence and leaf angle was nonlinear. In the presence of weeds, sugar beet had greater leaf angles (hyponastic; Figure 4). The inflection point for both early‐season removal and late‐season addition series occurred at 610 and 631 GDD, respectively, which is shortly before the leaf angles were measured (between 648 and 760 GDD). This suggests sugar beets can readily alter leaf angles in response to shade avoidance cues, and revert back quickly after the cue is removed.

Early‐season exposure to weeds had a season‐long impact on sugar beet leaf number and leaf area, even if the weeds were removed at the two true‐leaf stage (~330 GDD). Weeds present from sugar beet emergence to until the two true‐leaf stage reduced leaf number by 7% (25.8–23.9 leaves per plant; Figure 3c) and reduced leaf area by 22% (3,576 to 2,786 cm^2^; Figure 3e). Season‐long weed presence had no additional effect on sugar beet leaf area compared to the early‐season weed presence but reduced the number of leaves by an additional 10% compared to removal at the two true‐leaf timing (23.9–21.6 leaves per plant).

There was a positive linear relationship between the duration of weed‐free period and sugar beet leaf number and leaf area in the late‐season weed addition series (Figure 3d,f). Compared to the season‐long weed‐free treatment, weeds added at the sugar beet two true‐leaf stage (~330 GDD) and remaining until the end of the growing season (~1,240 GDD) reduced leaf number by 12% (25.5 to 22.4) and leaf area by 18% (3,555 to 2,929 cm^2^).

### 3.3 Season‐long shade avoidance has less effect on the earliest developing leaves compared to leaves that develop later

**FIGURE 5:**
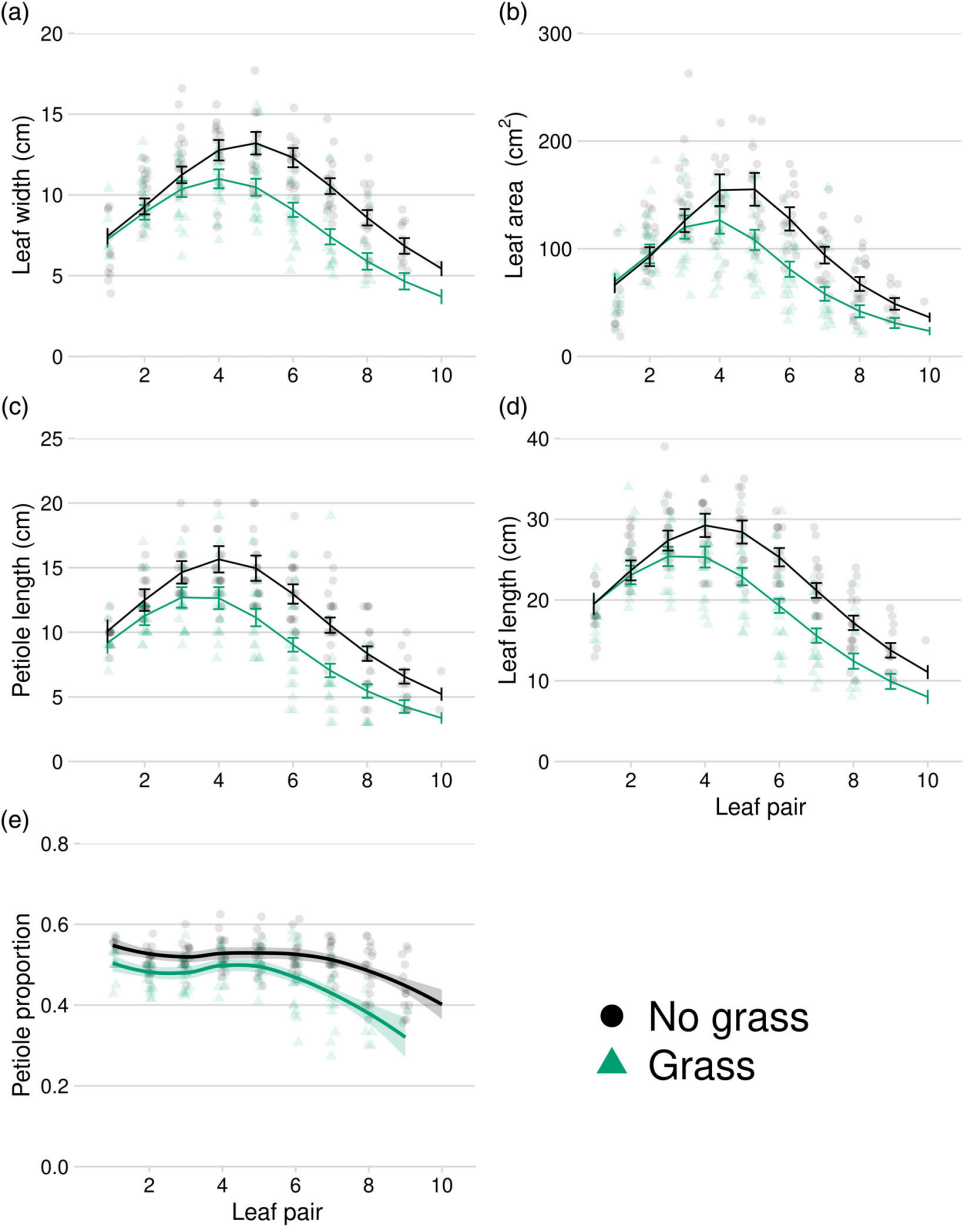
Relationship between sugar beet leaf position and leaf width (a), leaf area (b), petiole length (c), leaf length (d), petiole proportion of total leaf length (e) and lamina proportion of total leaf length (f) at harvest in a greenhouse study conducted from 11 Nov 2018, to 5 Feb 2019, Laramie WY. Leaf pair 1 indicates the oldest leaf pair [Colour figure can be viewed at wileyonlinelibrary.com]

In the greenhouse study, season‐long weed presence reduced sugar beet leaf width (Figure 5a), leaf area (Figure 5b), petiole and whole leaf length (Figure 5c,d), which confirmed the field study results. Differences in sugar beet leaf width, leaf area or total leaf length at the end of the study were not apparent on the oldest four leaves (leaf pairs 1 and 2) but were evident on the third and subsequent leaf pairs. Differences in petiole length between grass and no‐grass treatments were evident on nearly all leaves (Figure 5c). Petiole proportion (petiole length/whole leaf length) was greater in the weed‐free treatment for all leaf positions (Figure 5e), but the differences between treatments were more pronounced in the younger leaves (leaf pairs >5).

### 3.4 Early exposure to shade avoidance cues reduces leaf and root biomass production

In the greenhouse study, the presence of weeds at sugar beet emergence until harvest reduced leaf biomass by 35% (15.5–10.0 g, *p*‐value = .005) and root biomass by 32% (6.5–4.4 g, *p*‐value = .0002). The root to shoot biomass ratio was not different between weedy and weed‐free treatments (*p*‐value = .57). The leaf biomass reduction is a function of both fewer leaves developing (19.7 in the weed‐free treatment compared to 15.9 in the weedy treatment, *p*‐value = .0002), as well as smaller leaves (Figure 5). More leaves had senesced in the weed‐free treatment compared to the weedy treatment (2.8 and 1.7, respectively, *p*‐value = .02).

**FIGURE 6:**
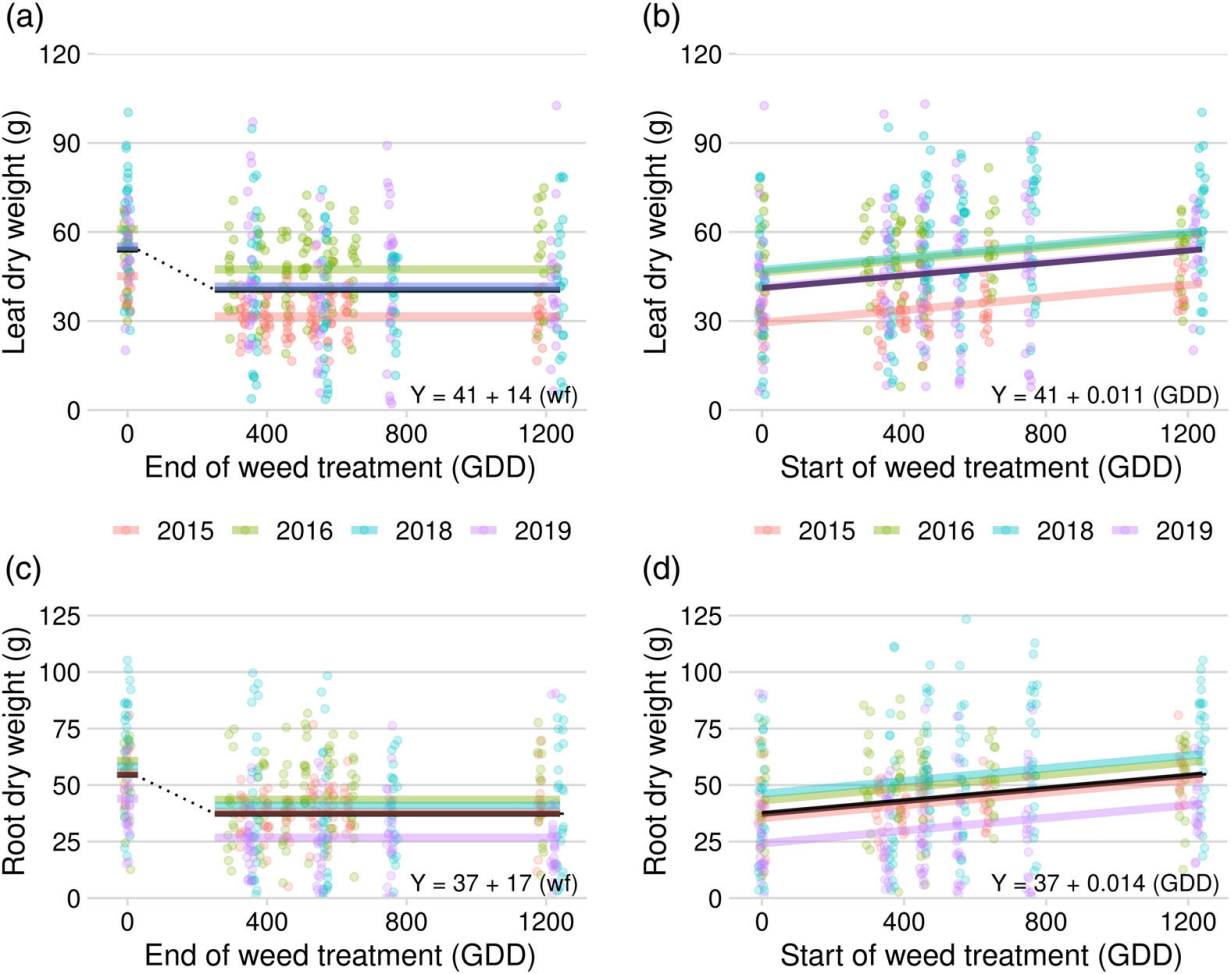
Relationship between duration of weed presence and sugar beet leaf biomass (a and b) and root biomass (c and d) in a field study conducted from 2015 to 2019, Laramie WY. Panels on the left (a and c) included treatments in which weeds were present at sugar beet emergence until removed later in the growing season (removal series). The dotted line in panels a and c indicates a break in the linear trend in the absence of the weed‐free treatment. Panels on the right (b and d) included treatments in which weeds were added at various times during the season and allowed to remain until harvest (addition series). Regression equation in each panel is for the fixed effects mean (black lines); ‘*wf*’ is an indicator variable for the weed‐free treatment (1 if weed‐free season‐long, 0 if weeds were present at any point during the season) [Colour figure can be viewed at wileyonlinelibrary.com]

In the field study, reduction in biomass in the early‐season removal series was entirely attributable to the early presence of the shade avoidance cues (*wf* indicator *p*‐value < .001). Table 2). Presence of weeds at sugar beet emergence reduced leaf and root biomass by 25% and 31%, respectively, even if weeds were removed at the two true‐leaf stage (~330 GDD) (Figure 6a,c). Longer duration of weed presence after the two true‐leaf stage did not reduce biomass further (*p*‐value >.4). In the late‐season series, when weeds were added at the sugar beet two true‐leaf stage and remained until harvest, sugar beet leaf biomass was reduced 18% and root biomass was reduced 23% compared to the weed‐free control (Figure 6b,d). The biomass lost from weed addition in the late‐season series followed a linear trend; for every 100 GDD that the sugar beets were kept weed‐free, root yield increased by 1.4 g compared to the season‐long weedy treatment (Figure 6d).

## 4 DISCUSSION

Results from this study showed that shade avoidance cues from early‐emerging weeds (weeds that emerge with the sugar beet) have a more detrimental impact on sugar beet growth and development than late‐emerging weeds. Sugar beet responded to an early shade avoidance cue by adjusting leaf orientation (hyponasty) to optimize light interception (Figures 3 and 4). When weeds were removed, the hyponastic response remained temporarily, but eventually reverted to a lower leaf angle. Shade avoidance cues also reduced the number of leaves and reduced leaf area for light capture and photosynthesis (Figure 3), thereby reducing shoot and root biomass (Figure 6). These results support our hypothesis and suggest a response where most, or even all, of the season‐long shade avoidance response (SAR) in sugar beet growth is fixed by the time the sugar beet crop has reached the two true‐leaf stage (approximately 330 GDD after planting). Removing weeds after that time had a small impact on the number of sugar beet leaves, but did not allow recovery of leaf area or biomass production, indicating that a long‐lasting shade avoidance response induced early in development may play a key role in crop yield loss due to weeds.

Studies have shown that stem and petiole extension and hyponasty are among the most common SARs (Cerrudo et al., 2017; Franklin & Whitelam, 2005; Yang & Li, 2017). Auxins play a key role in SAR (Sessa, Carabelli, Possenti, Morelli, & Ruberti, 2018). Along with brassinosteroids and cytokinins, auxin signalling results in leaf hyponasty, and stem and petiole elongation (Ruberti et al., 2012). Leaf orientation is an important factor influencing light interception in plants. This is because leaf angle relative to the direction of sunlight determines both the photosynthetic performance of the leaves at the top of the canopy as well as the amount of light available to lower leaves (Van Zanten, Pons, Janssen, Voesenek, & Peeters, 2010). At the canopy level, steeper leaf angles reduce mutual shading and thus, maximize light interception (Mullen, Weinig, & Hangarter, 2006). Since reduced R:FR is a cue for impending competition, it is not surprising that hyponasty is an intrinsic part of the shade avoidance syndrome (Van Zanten et al., 2010). Sugar beet has a rosette growth habit in the first season of growth and thus, hyponasty appears to be the strategy for projecting leaves for optimal light interception. We observed shade avoidance‐induced hyponasty in the form of increased leaf angles in our study (Figures 3 and 4); however, sugar beet did not increase petiole or leaf length in (Michaud, Fiorucci, Xenarios, & Fankhauser, 2017; Pantazopoulou et al., 2017, 2021) response to shade avoidance cues as might be expected. In fact, we observed a reduction in petiole length, total leaf length and petiole proportion of leaves in response to season‐long shade avoidance cues in our greenhouse study (Figure 5).

The lack of petiole or leaf length increase in sugar beet could due to the differences in PIF proteins between sugar beet and other plant species. For example, *A. thaliana* encodes 8 PIF proteins (PIF 1–8), while *B. vulgaris* only encodes homologs of *A. thaliana* PIFs 1, 3 and 7 (Casal, 2012; Dohm et al., 2014; Pham et al., 2018). In *A. thaliana* PIF4, PIF5 and PIF7 are the dominant PIF proteins involved in SAR (Hornitschek, Lorrain, Zoete, Michielin, & Fankhauser, 2009; Huber et al., 2021). All three PIF proteins (PIF4, PIF5 and PIF7) can bind to promoter regions of auxin‐associated genes but PIF7 plays a major role in the induction of auxin biosynthesis enzyme‐encoding YUCCA genes which stimulates auxin synthesis and transport from leaves to petioles to promote elongation and hyponasty under low R:FR (de Wit, Ljung, & Fankhauser, 2015; Michaud et al., 2017; Pantazopoulou et al., 2017, 2021). Under reduced R:FR light, PIF4 and PIF5 promote auxin responsiveness but PIF4, PIF5, and PIF7 act together regulate auxin production under reduced R:FR light (de Wit et al., 2016; Michaud et al., 2017; Pantazopoulou et al., 2017). Although PIF7 *A. thaliana* mutants do not display an elongated hypocotyl and petiole under low R:FR light, PIF 4 and 5 mutants still display a hypocotyl extension response to FR light, even if the effect is less pronounced (Hornitschek et al., 2012; Lorrain, Allen, Duek, Whitelam, & Fankhauser, 2008; Pantazopoulou et al., 2017). Leaf hyponasty and senescence still occur in the absence of these factors, implying important functional differences between sugar beet and Arabidopsis (Park et al., 2019; Sakuraba et al., 2014). Of the three PIF proteins *B. vulgaris* encodes, PIF3 seems to have the most functional overlap with the absent PIF homologues (Casal, 2012; Pham et al., 2018). Thus, PIF3 and PIF7 associated transcriptional changes may play major roles in responses described here.

Previous studies have found that reduced R:FR resulted in fewer leaves in *A. thaliana* (Xie et al., 2017) maize (Page, Liu, Cerrudo, Lee, & Swanton, 2011) and soybean (Green‐Tracewicz et al., 2012). We have previously shown that leaf number is reduced by season‐long shade avoidance cues in three different sub‐species of *B. vulgaris* (Schambow et al., 2019). Here, we show that although the first two leaf pairs were least affected with respect to leaf size and area (Figure 5), subsequent sugar beet leaf development was reduced substantially even if weeds were removed early in plant development (Figure 3). Although the physiological and molecular mechanisms of this inhibition of leaf production is not clearly established in sugar beet, studies on *A. thaliana* revealed that the activity Squamosa promoter binding protein‐like (SPL) genes non‐autonomously inhibit the initiation of new leaves at the apical meristem (Wang, Schwab, Czech, Mica, & Weigel, 2008). In addition, inactivation of the cytochrome P450 gene (CYP78A5), a gene that is expressed at the periphery of the apical meristem accelerated new leaf initiation rate. This suggests a potential compensatory role of SPL and CYP78A5 genes in modulating leaf initiation rate and leaf size in *A. thaliana* (Wang et al., 2008). In sugar beet, new leaves are produced from meristems which suggest that similar genes or orthologues could be involved in reducing new leaf production under weedy (far‐red light enriched) conditions.

Compared to season‐long weed‐free treatments in this study, the number of sugar beet leaves were reduced 10% if weeds were present for the first 16 days after emergence, and only reduced 12% if weeds were added 16 days after emergence and remained for the next 51 days (the average time from two true‐leaf stage to harvest in this study). Early‐emerging weeds had more impact on sugar beet leaf development compared to late‐emerging weeds.

This nearly‐irreversible effect of early‐emerging weeds on sugar beet could be due to a memory stress response, or a stress‐anticipatory response mechanism from very early stages of sugar beet development. Li, Swaminathan, and Hudson (2011) demonstrated that 1‐hr treatment of soybean seedlings with FR light resulted in distinctive light‐induced gene expression in the cotyledon, hypocotyl, shoot apical meristem and root phototropism gene (RPT2). Li et al. (2011) concluded that organ‐specific, light‐responsive transcriptional networks are active early in photomorphogenesis in dicotyledon seedlings. One possible response from such early exposure to stress is priming, where plants become more resistant to future exposure to such stressors by acquiring memory of the stress (Crisp, Ganguly, Eichten, Borevitz, & Pogson, 2016). This stress memory acquisition is modulated by several mechanisms and processes including RNA metabolism, posttranscriptional gene silencing and RNA‐directed DNA methylation (Crisp et al., 2016). For example, partial exposure of Arabidopsis leaves to high intensity light rapidly induced zinc finger transcription factor (*ZAT10*) mRNA in distal photosynthetic tissues, showing a possible preacclimation of photosynthetic tissues to high light (Rossel et al., 2007). However, in the case of shade avoidance, stress acclimation would have to be balanced against stress avoidance. Whether adaptive or maladaptive, there are costs to these memories, which might include repressed photosynthesis and reduced growth and development (Crisp et al., 2016; Soja, Eid, Gangl, & Redl, 1997). Thus, early exposure of sugar beet to reflected FR light could have resulted in maladaptive stress avoidance strategies (e.g., hyponasty and reduced leaf number) aimed at fortifying their defense against future light competition.

We have also observed that more leaves had senesced in the weed‐free treatment compared to the weedy treatment which contradicts previous observation that phytochrome mutants display PIFs‐mediated accelerated senescence (Sakuraba et al., 2014). The increased senescence observed in the weed‐free treatment was due to the increased rate of leaf production in the weed‐free treatment compared to the weedy treatment. Plants, in general, initiate senescence to shed photosynthetically ineffective leaves (Sakuraba et al., 2014). The phyllotaxy in sugar beet is such that as the plant produces more leaves, mutual shading of older leaves at the base of the plant intensifies. This reduces the photosynthetic efficiency of the older leaves which could explain the increased senescence in the treatment where more leaves were produced.

The relationship between sugar beet leaf area (Figure 3e,f) and the duration of weed presence follows a similar trend as number of leaves, with early presence of the shade avoidance cue initiating an irreversible season‐long response. Since leaf area is a function of leaf size and number, it appears the reduction in sugar beet leaf area was at least partly due to reduced number of leaves. Results from the greenhouse study showed that, in addition to reduced number of leaves, reflected FR light reduced sugar beet leaf width, petiole length and total leaf length as well (Figure 5). Differences in sugar beet leaf size were most pronounced after the first six true‐leaves had appeared, which corresponds to the transition from leaf canopy‐dominated growth to root cambium development (followed by storage root and sugar accumulation dominated phase) (Milford, 2006). Most capacity for sugar beet storage root size and weight is laid down by the sixth true‐leaf stage (Milford, 2006).

Although increased petiole elongation is a common response to shade avoidance cues (Weijschedé, Martínková, De Kroon, & Huber, 2006), we observed a reduction in petiole length (Figure 5c), even as a proportion of total leaf length (Figure 5e), from sugar beet plants exposed to weed‐reflected light in the greenhouse study. Sugar beet did not increase petiole length in response to shade avoidance cues as we had predicted based on previous work in other plant species. This illustrates the importance of conducting research on crop species with different growth habits, rather than generalizing from more commonly studied species like *Arabidopsis*, maize and soybean.

The reduction in sugar beet leaf number, leaf size and leaf biomass, likely contributed to reduced root biomass (Figure 6). It has been demonstrated that shade avoidance cues increase allocation to shoot growth at the expense of roots in *B. vulgaris* (Schambow et al., 2019). However, the mechanisms by which shade avoidance affects taproot development in root crops such as sugar beet is not well understood. Previous work has established that shade avoidance can affect root architecture, the formation of tubers and even the transcription of sugar transporting proteins essential in accumulating sucrose (Seabrook, 2005; van Gelderen, Kang, & Pierik, 2018). In potato (*Solanum tuberosum* L.), a root crop grown for its underground carbohydrate storage organ, tuberization and biomass allocation to roots is influenced by FR light through phytochrome B and transcription of sugar transporters (Chincinska et al., 2008; Seabrook, 2005).

It is clear from our results that weeds present at sugar beet emergence had a significantly greater impact on sugar beet growth compared to weeds added later in the season. Early weed removal is often recommended in sugar beets (Jalali & Salehi, 2013), because many previous studies have shown that early‐emerging weeds have a substantial effect on sugar beet yield; however, the mechanism driving those losses has not been characterized, and resource depletion has typically been assumed. Our results demonstrate that a substantial portion of reduced yield due to weeds may be due to shade avoidance, rather than solely resource depletion as is commonly thought. To protect sugar beet yield potential, ensuring a weed‐free environment at emergence may be critical.

It is important to state that crop yield loss due to shade avoidance is typically understood in the context of consistent FR light stimulation, but our understanding of shade avoidance responses at the molecular level is not well‐connected to whole‐plant responses in agronomically relevant contexts (Guo et al., 2017; Shikata et al., 2014). Alternative splicing occurs in response to FR light at the seed stage and changes development into the seedling stage, but the effects on further growth are not well understood after this point (Penfield, Josse, & Halliday, 2010; Shikata et al., 2014). There is evidence that PIF proteins are upregulated specifically in response to weed competition, although these do not occur in all species (Horvath et al., 2015, 2019; Page, Tollenaar, Lee, Lukens, & Swanton, 2009). Previous studies have shown that microRNAs (MIR156s), together with SPL genes that affect diverse plant developmental processes such as leaf development, are also involved in growth‐phase transition, and thus, can affect organ size and whole‐plant biomass (Fu et al., 2012; Toriba et al., 2019; Wang et al., 2008; Wu et al., 2009; Xie et al., 2017). This latter observation is particularly intriguing in light of differential leaf area development shown in Figure 3; that a shade avoidance response triggered so early in development could play a significant role in crop yield loss opens a potential pathway for improved breeding and bioengineering strategies.

We conclude that sugar beet exhibits a shade avoidance response when exposed to early‐emerging weeds that causes season‐long impacts that affecting growth, development, and yield potential. Thus, this work has significant practical implications for weed management (e.g., timing of weed removal) as well as crop genetic improvement. However, our study system ensured a strong reflected R:FR light ratio at the time of sugar beet emergence, and the weed density or proximity of weeds to the sugar beet plants required to cause a similar response under more typical field conditions remains unknown. This will no doubt vary by weed species (e.g., differences in reflectance, growth habit [erect vs. prostrate], etc.), crop row spacing and other agronomic factors. Although it is uncommon for weeds to be extremely dense in the field at the time of sugar beet emergence, there is growing interest in ‘planting green’, where a cash crop is seeded directly into established cover crops, and this work suggests that practice may have unforeseen impacts on crop yield potential.

## CONFLICT OF INTEREST

The authors have no conflict of interest to declare.

## Data Availability

The data that support the findings of this study are openly available at figshare: https://doi.org/10.6084/m9.figshare.15170457.
